# Expression of calcineurin, calpastatin and heat shock proteins during ischemia and reperfusion

**DOI:** 10.1016/j.bbrep.2015.09.016

**Published:** 2015-09-25

**Authors:** Sreejit Parameswaran, Rajendra K. Sharma

**Affiliations:** Department of Pathology and Laboratory Medicine, College of Medicine, University of Saskatchewan, Saskatoon, Saskatchewan, Canada S7N 5E5

**Keywords:** Calpn, calpain, CaN, calcineurin, Calp, Calpastatin, HMWCaMBP, high molecular weight calmodulin-binding protein, NMCC, primary neonatal mouse cardiomyocyte culture, I/R, Ischemia and Reperfusion, NDB, nutrient deficient buffer, FACS, flow cytometry, FITC, fluorescein isothiocyanate, PE, R-phycoerythrin, Primary cardiomyocyte culture, Ischemia, Reperfusion, Calcineurin, Calpastatin, Heat shock proteins

## Abstract

**Objective:**

Calcineurin (CaN) interacts with calpains (Calpn) and causes cellular damage eventually leading to cell death. Calpastatin (Calp) is a specific Calpn inhibitor, along with CaN stimulation has been implicated in reduced cell death and self-repair. Molecular chaperones, heat shock proteins (Hsp70 and Hsp90) acts as regulators in Calpn signaling. This study aims to elucidate the role of CaN, Calp and Hsps during induced ischemia and reperfusion in primary cardiomyocyte cultures (murine).

**Methods and results:**

Protein expression was analyzed concurrently with viability using flow cytometry (FACS) in ischemia- and reperfusion-induced murine cardiomyocyte cultures. The expression of Hsp70 and Hsp90, both being molecular chaperones, increased during ischemia with a concurrent increase in death of cells expressing these proteins. The relative expression of Hsp70 and Hsp90 during ischemia with respect to CaN was enhanced in comparison to Calp. Reperfusion slightly decreased the number of cells expressing these chaperones. There was no increase in death of cells co-expressing Hsp70 and Hsp90 along with CaN and Calp. CaN expression peaked during ischemia and subsequent reperfusion reduced its expression and cell death. Calp expression increased both during ischemia and subsequent reperfusion but cell death decreased during reperfusion.

**Conclusion:**

The present study adds to the existing knowledge that Hsp70, Hsp90, CaN and Calp interact with each other and play significant role in cardio protection.

## Highlights

• Differential expression of calcineurin and calpastatin during ischemia and reperfusion.
• Enhanced ischemia induced cell death in cells expressing Hsp70 and Hsp90.
• Cardio protective role of calcineurin, calpastatin, Hsp70 and Hsp90.

## 1 Introduction

The increase in Ca^2+^ concentration during ischemia causes activation of calpains (Calpn) [1]. Calpn activation results in protein degradation and cell death [2], [3]. Calpn activation has been well studied in normal and ischemic cardiomyocytes [1], [4]. Cells at the ischemic infarct edge, which have undergone partial ischemia are also vulnerable to remodeling [5]. Due to impaired intracellular Ca^2+^ homeostasis, such cells are predisposed to death following reperfusion [6]. Interestingly, cardiomyocyte proliferation and progenitor cell recruitment has been observed in the cardiac infarct border zone [7]. Calpastatin (Calp) is the most efficient and specific Calpn inhibitor present *in vivo* [8], [9], [10]. Calp along with its putative homolog high molecular weight calmodulin-binding protein (HMWCaMBP) regulate Calpn inhibition [11], [12], [13], [14], [15] and may reduce I/R injury in heart [16], [17].

Among the proteins proteolysed by Calpn, calcineurin (CaN) is known to regulate cardiac hypertrophy and remodeling and has been implicated in both cell death and survival following reperfusion [18], [19], [20]. CaN is a heterodimer consisting of 19- and 57-59- kDa subunits which are referred to as CaNβ and CaNα, respectively [21], [22], [23]. The CaNα subunit has low endogenous phosphatase activity and requires Ca^2+^, calmodulin (CaM) and CaNβ for full activity [24]. CaN activation during ischemia occurs due to elevated Calpn levels [13], [25], [26], [27] which has been demonstrated through *in vitro* proteolytic degradation [28] or via the cleavage of the endogenous calcineurin inhibitor cain/cabin1 [29]. Recent studies propose that ischemia induced activation of CaN leads to further increase in cytosolic Ca^2+^ levels, which further activates Calpn during reperfusion [30]. This putative feedback mechanism can influence CaN–Calpn signaling in cardiomyocytes following ischemia and reperfusion (I/R) [1], [26]. Interestingly, the CaM-dependent phosphatase activity of CaN is stimulated by the 70 kDa heat-shock protein (Hsp70) in cardiac muscle and thus provides an on/off switch for the regulation of CaN signaling by Hsp70 [31]. CaN–Hsp70 signaling results in the activation of NFAT which affects apoptosis, development and cellular adaptation in cardiac cells [31], [32], [33]. The importance of CaN–Hsp70 interaction lies with downstream effectors such as NFAT and GATA-4, which are important in cardiac remodeling and regeneration [34], [35], [36].

Recently, Hsp90, another heat-shock protein, has generated attention due to its cardiac protective role in I/R induced injury [37], [38], [39], [40], [41]. In septic mice models, Calpn induces caspase-3 activation and apoptosis via the activation of the Hsp90/Akt pathway [42]; however, this activation can also promote CaN recruitment to prevent apoptosis [43], [44]. Hsp90 also plays an important role in regulating Calpn-1 through specific interactions and associations at the functional sites. Nevertheless, Hsp90 can get degraded in concentrations higher than equimolar levels of Calpn [45]. Though both Hsp70 and Hsp90 are molecular chaperones [46] and appear to have cardioprotective properties, several differences exist especially at mRNA induction during I/R [47], [48], [49], [50], [51]. The interaction and the relevance of Hsp70 and Hsp90 in I/R with respect to Calpn-regulated proteins like CaN and Calp remains vague. The current study aims to reveal the underlying interplay of CaN, Calp, Hsp70 and Hsp90 during ischemia and subsequent reperfusion using flow cytometric analysis (FACS). The expression level of ubiquitous cardiac protein sarcomeric actin (SarcAct) has been also studied as a control.

## 2 Methodology

### 2.1 Cells

Neonatal murine cardiomyocyte culture (NMCC – primary cultures derived from isolated murine heart) was used for studying the induced I/R injury. CD-1 Swiss albino mice pups (2–6- day old) were sacrificed, in accordance to the norms provided by the Institutional Animal Ethics Committee, University of Saskatchewan. The hearts were instantly extracted, processed and cultured on 0.02% gelatin-precoated cell culture flasks, based on protocols previously described [52], [53]. The primary cultures were sustained till the cultures attained ~80% and following which I/R injury was induced in cell cultures.

### 2.2 I/R injury induction

The media in NMCC cultures (~80% confluent) was replaced 24 h preceding induction. Ischemic conditions were induced by replacing the standard growth media with a nutrient deficient buffer (NDB). The NDB contains 136 mM NaCl, 5 mM KCl, 1 mM CaCl_2_, 0.5 mM MgCl_2_·7H_2_O and 5.5 mM HEPES (pH 6.8) and therefore provides no nutrition and minimal buffering to the cells [54]. For inducing ischemia in NMCC, glucose and FCS were added to NDB to obtain a final concentration of 5 mM and 2%, respectively to provide basic minimal nutrition [55]. Consecutively, reperfusion was performed by switching NDB with standard growth media [54], [55], [56]. In addition, to emulate the oxidative stress in cardiomyocytes observed *in vivo* during reperfusion, hydrogen peroxide (H_2_O_2_) was added to the standard growth media (1 mM final concentration) [54], [55]. The methodology was performed as per a previously published protocol [13], [14].

### 2.3 Assessment of protein expression and viability

The concurrent assessment of protein expression in normal (untreated), ischemic and reperfused cardiomyocytes along with viability was performed by FACS based on a methodology carried out as per a previously published protocol [13], [14]. Briefly, the assay of live versus dead cells was used to assess viability following induction and compared to control cells. The assay was performed simultaneously with FACS analysis using 7-amino-actinomycin D (7-AAD) [57]. As suggested by the manufacturer, 7-AAD staining solution in DPBS (~0.25 μg/10^6^ cells) was incubated with control, ischemia and reperfusion induced cells for 10 min at room temperature in the dark. The cells were washed twice with DPBS and dislodging for FACS. The ideal I/R injury induction was determined by inducing the cells at different parameters (ischemia induction – 1, 2 and 4 h; reperfusion induction following ischemia – 1 and 2 h). The induced cells along with control cells were stained with 7-AAD and dislodged by trypsinization. The cell suspension was immediately used for FACS to quantify live and dead cells in the control and induced population. A tabulation of antibodies along with the dilutions used are provided in Supplementary Table 1.

### 2.4 Statistical analysis

Statistical analysis on the data obtained from the various assays was performed using ANOVA (Sigma Plot version 10 software package). The significance level of ≤0.05 is represented as * to indicate significant differences.

## 3 Results and discussion

**Fig. 1:**
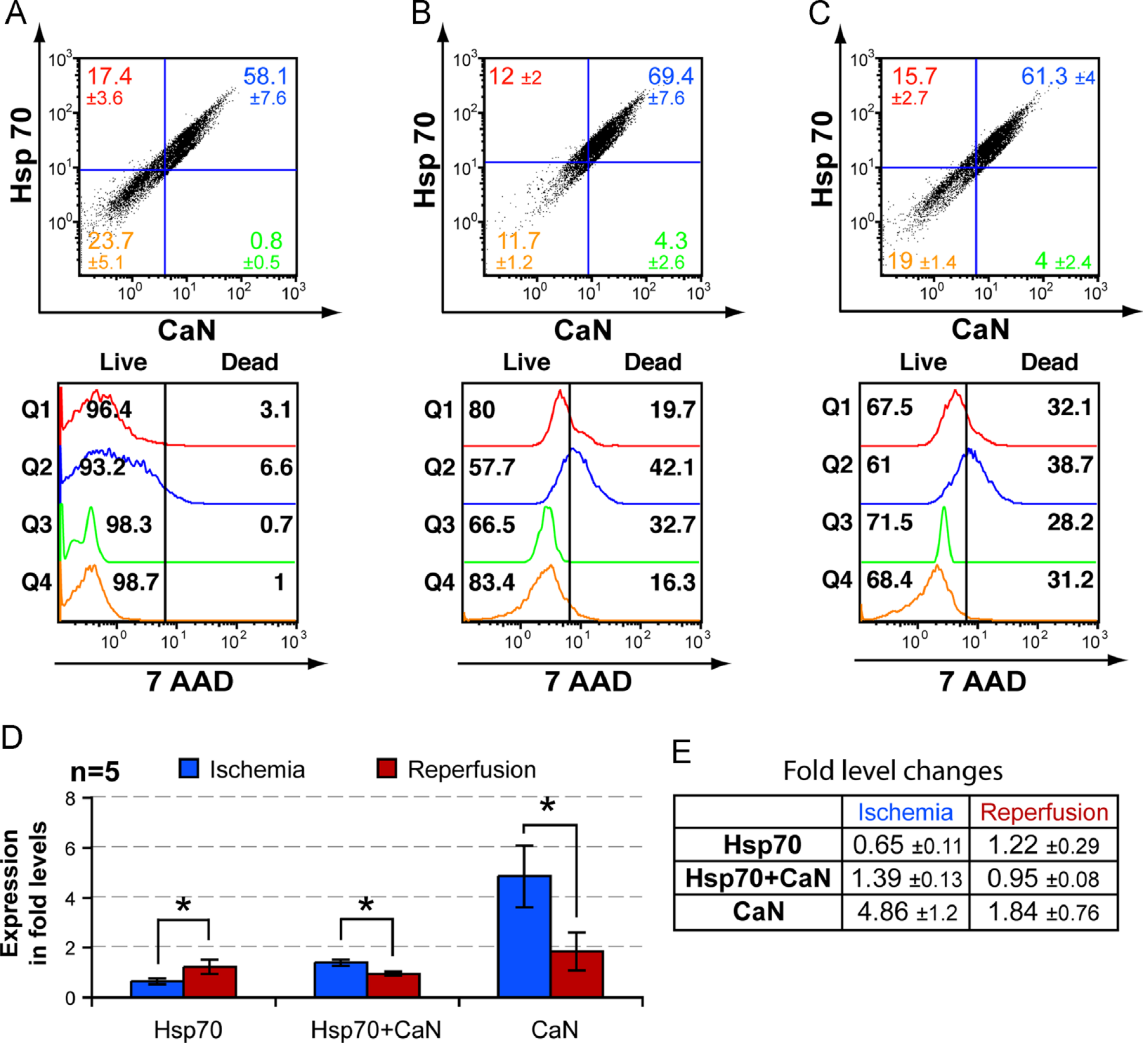
(A)–(C) Representative FACS analysis data of NMCC following I/R induction along with live–dead assay. In the horizontal axis FITC labeled anti-CaN antibodies and for vertical axis PE labeled antibodies against Hsp70 were detected. Rest of the figures in the panel are derived from the quadrants of (A)–(C) and demonstrate the live–dead assay using 7-AAD. The studied conditions were; normal untreated NMCC (A); NMCC maintained in nutrient deficient buffer (ischemia induction) for 2 h (B); NMCC grown for 2 h in standard growth media containing 1 mM H_2_O_2_ subsequent to 2 h of ischemia induction (reperfusion induction) (C). (D) Histographical representation of comparative protein expression in ischemia and reperfusion induced NMCC with those of normal untreated NMCC within stained quadrants (Q1 – Hsp70; Q2 – Hsp70+CaN; Q3 – CaN) represented as fold level change (*n*=5). The fold level changes (increase or decrease) of protein expressing NMCC in each quadrant has been represented and significant values (*p*-value<0.05) denoted as *. Standard error was calculated and represented as error bars. (E) Fold level changes in ischemia and reperfusion induced protein expression in NMCC within stained quadrants (Q1–Q3) in comparison with control cells (*n*=5) represented as a table.

**Fig. 2:**
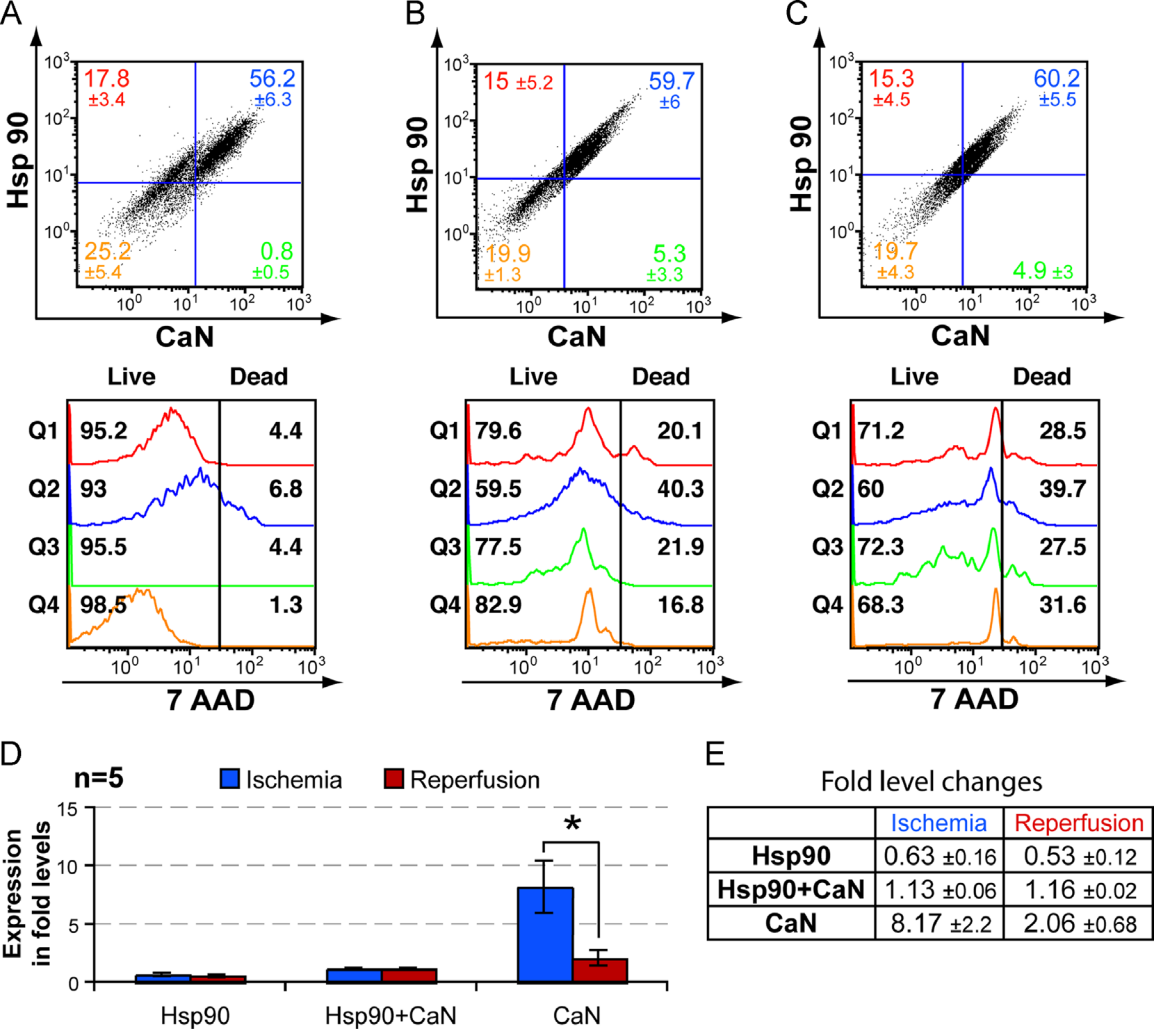
(A)–(C) Representative FACS analysis data of NMCC following I/R induction along with live–dead assay. In the horizontal axis FITC labeled anti-CaN antibodies and for vertical axis PE labeled antibodies against Hsp90 were detected. Rest of the figures in the panel are derived from the quadrants of (A)–(C) and demonstrate the live–dead assay using 7-AAD. The studied conditions were; normal untreated NMCC (A); NMCC maintained in nutrient deficient buffer (ischemia induction) for 2 h (B); NMCC grown for 2 h in standard growth media containing 1 mM H_2_O_2_ subsequent to 2 h of ischemia induction (reperfusion induction) (C). (D) Histographical representation of comparative protein expression in ischemia and reperfusion induced NMCC with those of normal untreated NMCC within stained quadrants (Q1 – Hsp90; Q2 – Hsp90+CaN; Q3 – CaN) represented as fold level change (*n*=5). The fold level changes (increase or decrease) of protein expressing NMCC in each quadrant has been represented and significant values (*p-* value<0.05) denoted as *. Standard error was calculated and represented as error bars. (E) Fold level changes in ischemia and reperfusion induced protein expression in NMCC within stained quadrants (Q1–Q3) in comparison with control cells (*n*=5) represented as a table.

**Fig. 3:**
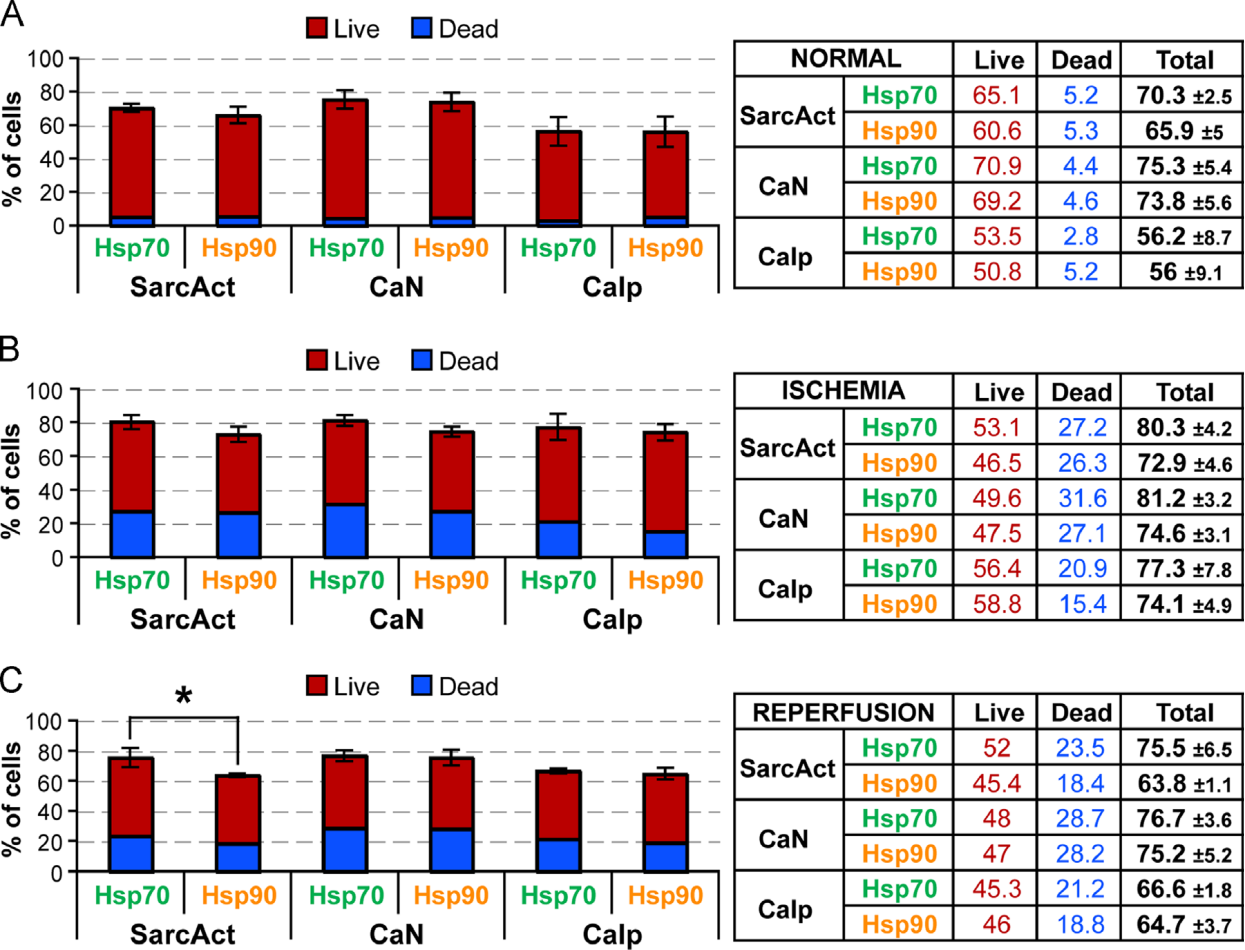
Tabulated representation of percentage of cells expressing Hsp70 and Hsp90 relative to SarcAct, CaN and Calp under different conditions. The study conditions used were; normal untreated NMCC (A); NMCC treated with nutrient deficient buffer (ischemia induction) for 2 h (B); NMCC grown for 2 h in normal media containing 1 mM H_2_O_2_ following 2 h of ischemia induction (reperfusion induction) (C). The averaged live and dead cell percentages was used to calculate the percentage of cells expressing Hsp70 or Hsp90 only or with cells co-expressing Hsp70 or Hsp90 with SarcAct or CaN or Calp. The total percentage of cells was then determined by adding the percentage of cells expressing Hsp70 or Hsp90 only, along with cells co-expressing Hsp70 or Hsp90 with SarcAct or CaN or Calp. Significant values (*p*-value<0.05) denoted as * was determined. Standard error was calculated and represented as error bars.

The triple staining was performed by concurrently staining two proteins with specific antibodies tagged with fluorophore (FITC and PE, respectively) along with a live–dead assay of analyzed cells with 7-AAD. The analysis elucidated the expression of various cardiac proteins in both live and dead cells present in control and I/R treated cardiomyocyte cultures. This differentiation quantified cells which survived I/R injury and determined the important proteins expressed in cells [13], [14]. In the present study, the interaction of Hsp70 and Hsp90 with respect to CaN (Figs. 1 and 2) was compared to Calp (Supplementary Figs. 1 and 2). The expression levels (as percentages and fold levels) were also compared in a control protein which is ubiquitously expressed (SarcAct) (Fig. 3, Supplementary Figs. 3 and 4).

### 3.1 Expression of Hsp70 and Hsp90 in CaN expressing cells

On comparing Hsp70 with CaN in normal, ischemia induced and reperfusion induced cells, we observed a global increase in expression of both Hsp70 and CaN following ischemia which significantly decreased following reperfusion (Figs. 1 and 3). Cardiomyocytes expressing both Hsp70 and CaN peaked during ischemia and then significantly decreased during reperfusion (Fig. 1D). A slight increase in global expression of Hsp90 with respect to CaN following ischemia and subsequent reperfusion was observed but not significant (Fig. 3). Conversely, the number of cells expressing Hsp90 alone decreased during both ischemia and reperfusion, whereas there was slight increase in cells co-expressing Hsp90 and CaN during ischemia and subsequent reperfusion (Fig. 2). There was simultaneous increase in number of dead cells expressing Hsp70 or Hsp90 during ischemia (Figs. 1B and 2B). The drastic increase in dead cells co-expressing Hsp70 or Hsp90 and CaN during ischemia was not observed during subsequent reperfusion (Figs. 1C and 2C).

The current study clearly shows that the stress induced by ischemic treatment simultaneously increased the expression of Hsp70 and Hsp90 since both being chaperone proteins [46]. The cells expressing Hsp70 and Hsp90 predominantly died during ischemia (Figs. 1B and 2B) [50]. Reperfusion did not enhance cell death indicating that the cells expressing Hsp70 or Hsp90 were protected against subsequent reperfusion induced injury (Figs. 1C and 2C). Activation of Heat Shock Transcription Factor 1 during ischemia stimulates Hsp70 mRNA expression whereas reperfusion stimulates Hsp90 mRNA [51]. The marked increase observed in Hsp70 expression in CaN expressing cells (Fig. 1B) suggests the possible interaction between Hsp70 and CaN as previously described [13], [31]. Hsp70 phosphorylation by cAMP-dependent protein kinase (PKA) (produced in conditions of stress), can inhibit Hsps ability to enhance CaN phosphatase activity [31], [58]. It is possible that the cells try to compensate the loss of enhanced CaN phosphatase activity by producing more CaN and hence the increased CaN expression during ischemia (Fig. 1D and E). The interaction between Hsp70 and CaN is known to dephosphorylate NFAT resulting in transcriptional induction of various genes including GATA4, which is crucial in cardiomyocyte development and has a significant role in unassisted self-repair [32], [36]. Conversely, ischemia induced CaN dephosphorylates phospholamban which results in Ca^2+^ overload during reperfusion and thus further damages the cells [30]. It is also known that increase in myocardial Hsp90 expression promotes the recruitment of Akt and CaN, thereby promoting endothelial nitric oxide synthase (eNOS) activation and subsequently reducing cell injury [43], [44]. Therefore, it can be assumed that the slight increase in cells co-expressing Hsp90 and CaN (Fig. 2D and E), stimulated eNOS production which resulted in no further increase in cell death during reperfusion.

A significant increase in cardiomyocytes expressing CaN only with respect to both Hsp70 and Hsp90 was observed following ischemia which then significantly reduced during reperfusion (Figs. 1D and 2D). The findings on the expression of CaN were similar to our previous study in human and animal heart [59]. Interestingly, the percentage of CaN expressing dead cells increased following ischemia and subsequent reperfusion (Figs. 1E and 2E), compared to our previous report where the percentages of CaNα and CaNβ subunit expressing dead cells decreased during ischemia and subsequent reperfusion [13]. Both CaNα and CaNβ subunits are required for the phosphatase activity of CaN along with CaM and Ca^2+^ [24]. CaNα and CaNβ subunits are more apparent in live cells in comparison to dead cells where these subunits may be completely proteolysed by Calpn [28]. Expression of CaN alone in cells increased by ~4 and ~8 fold with respect to Hsp70 and Hsp90 respectively during ischemia (Figs. 1E and 2E). The absence of Hsp70 and Hsp90 greatly increased cell death and decreased CaN expression which could be due increased Calpn production (Figs. 1B and 2B). Reperfusion further decreased CaN expression and there was no significant increase in number of dead cells compared to ischemic induction in absence of Hsp70 (Figs. 1C and 2C). Absence of both Hsp70 and Hsp90 in cells expressing CaN only could be attributed to the proteolytic activity of Calpn [25], [26], [27], [45], [60]. The cardiomyocytes in such conditions try to overcome the potential cell damage by increasing CaN expression and activation during ischemia as previously reported [13], [27]. Reperfusion decreases the overall Calpn expression which results in decreased CaN expression and activation [13].

### 3.2 Expression of Heat Shock Protein (Hsp) 70 and Hsp90 in Calpastatin (Calp) expressing cells

In normal (untreated), ischemia induced and reperfusion induced cardiomyocytes on comparing with Calp, we observed a global increase in expression of both Hsp70 and Hsp90 following ischemia which significantly decreased following reperfusion (Fig. 3, Supplementary Figs. 1 and 2). Cardiomyocytes expressing Hsp70 only slightly decreased during ischemia and showed increase in number of dead cells (Supplementary Fig. 1B). Subsequent reperfusion did not increase the expression of Hsp70 as the number of dead cells increased significantly (Supplementary Fig. 1C). The cardiomyocytes expressing both Hsp70 and Calp increased slightly during ischemia with significant increase in dead cells (Supplementary Fig. 1D and E). Reperfusion further increased the number of dead cells co-expressing both Hsp70 and Calp (Supplementary Fig. 1C). Increased expression of Hsp90 in cardiomyocyte following ischemia and subsequent reperfusion was observed (Supplementary Fig. 2). However, the number of cells co-expressing Hsp90 and Calp remained same as untreated cells during ischemia with a considerable increase in dead cells (Supplementary Fig. 2B). A slight increase in number of cells co-expressing Hsp90 and Calp was observed during subsequent reperfusion along with no significant increase in dead cells (Supplementary Fig. 2C). Ischemia enhanced the relative expression of Hsp70 and Hsp90 in cardiomyocytes with respect to Calp as compared to CaN. The increase in expression is related to the increase in number of live cells expressing Hsp70 and Hsp90 expression was much higher than the increase in number of live cells (Fig. 3B). Reperfusion produced a decrease in number of live cells expressing Hsp70 and Hsp90 with a slight increase in number of dead cells (Fig. 3C).

The overall expression of Calp increased during ischemia and subsequent reperfusion (Supplementary Figs. 1 and 2). The expression of Calp marginally increased during ischemia and subsequent reperfusion in cardiomyocytes co-expressing Hsp70 (Supplementary Fig. 1B and D). There was an increase in number of dead cells during ischemia, and subsequent reperfusion did not increase the number of dead cells. The number of Calp only producing cells relative to Hsp70 did not increase much during ischemia though the death rate was considerably increased (Supplementary Fig. 1D). Subsequent reperfusion induced a significant increase (~4 fold) in number of Calp only producing cardiomyocytes with a significant increase in number of dead cells than in ischemia.

The expression of Calp barely increased during ischemia and subsequent reperfusion in cardiomyocytes co-expressing Hsp90 (Supplementary Fig. 2D and E). As with Hsp70 co-expression, the number of dead cells increased during ischemia and persisted during subsequent reperfusion. The number of Calp only producing cells relative to Hsp90 slightly increased during ischemia with increase in number of dead cells (Supplementary Fig. 2B). Reperfusion induced a slight increase in number of Calp only producing cardiomyocytes with a decrease in number of dead cells than in ischemia.

Ischemic treatment increased the expression of Hsp70 and Hsp90 since both are chaperone proteins (Supplementary Figs. 1B and 2B). However, a significant number of cells co-expressing Hsp70 or Hsp90 with Calp died during ischemia. Reperfusion significantly enhanced the killing of cells expressing Hsp70 or Hsp90 only compared to those co-expressing Calp with Hsp70 or Hsp90. This suggests that the expression of Calp also protected cardiomyocytes against reperfusion induced injury. Calp sequesters Calpn from its substrates in the normal myocardium, but may be proteolysed during the early phase of Calpn activation during I/R [12], [14]. Calpn activation results in the proteolysis of Calp followed by other calpain substrates [61]. It is known that Calpn cleaves in Hsp70 during neuronal degradation [60]. It is therefore possible that the absence of Hsp70 in Calp only expressing cardiomyocytes makes them more susceptible to death. An increase in number of Calp only expressing dead cells relative to Hsp70 suggests that Hsp70 plays a critical role in cardioprotection. On the other hand, the decrease in number of Calp only expressing dead cells relative to Hsp90 during reperfusion is significant such that the levels return to almost normal (untreated). Hsp90 has both cardioprotective and antagonistic characteristics [37], [38], [39], [40], [41], [42], [45]. Absence of Hsp90 occurs during ischemia, makes Calp more susceptible to Calpn degradation. Calpn activity can be regulated by pathways other than AKT also since Calpn can degrade AKT-associated Hsp90 [27], [42]. In such context, it can be inferred that Calpn can proteolyse Hsp90 and Calp and killed the cardiomyocytes and in these dead cells neither Hsp90 nor Calp expression could be detected. It should be noted that there was a concurrent and drastic increase in number of dead cells following reperfusion which did not express Hsp90 or Calp.

### 3.3 Expression of Hsp70 and Hsp90 in SarcAct expressing cells

On comparing with SarcAct, we observed a global increase in expression of both Hsp70 and Hsp90 following ischemia which significantly decreased following reperfusion (Fig. 3, Supplementary Figs. 3 and 4). Non-cardiomyocytes expressing Hsp70 only significantly increased during ischemia and showed increase in number of dead cells (Supplementary Fig. 3B). Subsequent reperfusion did not increase the number of dead cells but reduced the number of Hsp70 expressing cells (Supplementary Fig. 3C). The cardiomyocytes expressing both Hsp70 and SarcAct remained consistent during ischemia with significant increase in dead cells (Supplementary Fig. 3D). Reperfusion further increased the number of dead cells co-expressing both Hsp70 and Calp but the expression of Hsp70 remained same. Increased expression of Hsp90 alone in non-cardiomyocytes following ischemia was observed with increased cell death (Supplementary Fig. 4B). Subsequent reperfusion decreased Hsp90 only expression in cells without any increase in dead cells (Supplementary Fig. 4C). However, the number of cells co-expressing Hsp90 and SarcAct remained same as untreated cells during ischemia with a considerable increase in dead cells (Supplementary Fig. 4D and E). The number of cells co-expressing Hsp90 and SarcAct remained consistent during subsequent reperfusion along with no significant increase in dead cells. Ischemia slightly decreased the number of cardiomyocytes expressing SarcAct only with respect to Hsp70 but with a marginal increase in cell death. Subsequent reperfusion produced no change in number of cardiomyocytes expressing SarcAct only with respect to Hsp70 with no change in number of dead cells (Supplementary Fig. 3). With respect to Hsp90, number of cells expressing SarcAct only, decreased during ischemia with no significant increase in cell death (Supplementary Fig. 4). Ensuing reperfusion also induced a decrease in number of cells expressing SarcAct only with respect to Hsp90 but with significant increase in cell death.

It is evident from the experiments in this study using CaN and Calp that ischemia induces an overall increase in expression of Hsp70 and Hsp90 (Fig. 3) and this trend was observed when Hsp70 and Hsp90 expression was measured relative to SarcAct. A comparable trend was observed during reperfusion where the expression of Hsp70 and Hsp90 decreased slightly but still more than normal levels. SarcAct is ubiquitous and is not affected by the changes in the proteins studied in this study (Hsp70, Hsp90, CaN, Calp). It is known that sarcomeric proteolysis via calpain and caspase activation may be involved to cooperatively degrade proteins including myosin, actin, troponin, and tropomyosin [62], [63]. Our previous studies using Calpn [13], [14] demonstrated the interaction between Calpn and other CaM-regulated proteins. Since Calpn has the potential to degrade SarcAct, we did not use the Calpn to avoid any confusion, as we intended to compare the expression of other proteins used in this study with a protein which is integral and consistently present in cardiomyocytes. Therefore cells which did not express SarcAct were considered as non-cardiomyocyte population which includes fibroblast, macrophages, and other cells present cardiac tissue [64]. The expression of SarcAct was observed to be consistently about ~60% of the total cells. Compared to SarcAct, the expression of other proteins varied depending on the treatment and interactions. In relation to Hsp70, the reduction of SarcAct only expressing cells suggests that the cells started co-expressing Hsp70 during both ischemia and reperfusion (Supplementary Fig. 3D). The rise in SarcAct only expressing cells in relation to Hsp90 during reperfusion (Supplementary Fig. 4D) suggests reduction in expression of Hsp90 which correlates to a simultaneous decrease in Hsp90 expression in non-cardiomyocyte population.

A semi-quantitative estimation of Hsp70, Hsp90, CaN, Calp and SarcAct levels in cells was determined by Western blotting. The results obtained showed insignificant changes in the expression levels in proteins studied (data not shown). The absence of currently used methodologies or technologies to determine the expression level of proteins simultaneously in live and dead cells, other than FACS, hinders data validation. Repetition of experiments is the only proof of evidence in this circumstance. The drawback of the potential loss of floating dead cells which is often discarded during the washing steps has been negated in our previous studies [13], [14], by the adding the pellets of pooled media or buffering solution discarded after cell treatments.

This is the first report which compares the expression of CaN, Calp and Hsps (70 and 90) in cardiomyocytes during ischemia and subsequent reperfusion. Previous studies were only able to gauge the global expression of these proteins in cardiomyocytes without any distinction of being live or dead [12], [59], [65], [66], [67]. The current study uses FACS based assay to differentiate live and dead cells and also quantifies the protein expression in these cells separately. Further studies using animal knockdown models and rescue assays using over-expressed proteins can support these novel findings.

In brief, the present study describes the use of triple staining for comparative protein expression analysis of Hsp70, Hsp90, CaN, Calp and SarcAct in normal, ischemia-induced and reperfused cardiomyocytes by FACS. Ischemia induces an increase in the expression of molecular chaperones Hsp70 and Hsp90 in cardiomyocytes along with increase in cell death. The expression of Hsp70 and Hsp90 decreases slightly during reperfusion. The absence of enhanced cell death suggests the cardioprotective nature of these proteins (Hsp70 and Hsp90). CaN expression peaks during ischemia and reduces during subsequent reperfusion similar to our previous studies [13]. An increase in cell death was observed in cells expressing CaN following ischemia but no further increase in cell death during reperfusion implies that the CaN expression promotes cell survival and is therefore cardioprotective. Calp expression increased during ischemia and subsequent reperfusion much similar to previous reports [13], [14]. Decreased cell death was observed in cells co-expressing Calp with Hsp70 and Hsp90 during reperfusion compared to cells expressing only hsp70 or Hsp90 or Calp. This suggests the cardioprotective role of Calp by inhibiting Calpn. Expression of SarcAct remained consistent in cardiomyocytes since being ubiquitous and used as a control. Thus this study validates the cardioprotective nature of Hsp70, Hsp90, CaN and Calp previously reported by many groups.

## Appendix A Supplementary material

Supplementary material Supplementary Fig. 1**.** (A)–(C) Representative FACS analysis data of NMCC following I/R induction along with live-dead assay. In the horizontal axis FITC labeled anti-Calp antibodies and for vertical axis PE labeled antibodies against Hsp70 were detected. Rest of the figures in the panel are derived from the quadrants of (A)–(C) and demonstrate the live-dead assay using 7-AAD. The studied conditions were; normal untreated NMCC (A); NMCC maintained in nutrient deficient buffer (ischemia induction) for 2 h (B); NMCC grown for 2 h in standard growth media containing 1 mM H_2_O_2_ subsequent to 2 h of ischemia induction (reperfusion induction) (C). (D) Histographical representation of comparative protein expression in ischemia and reperfusion induced NMCC with those of normal untreated NMCC within stained quadrants (Q1 – Hsp70; Q2 – Hsp70+Calp; Q3 – Calp) represented as fold level change (*n*=4). The fold level changes (increase or decrease) of protein expressing NMCC in each quadrant has been represented and significant values (*p*-value<0.05) denoted as *****. Standard error was calculated and represented as error bars. (E) Fold level changes in ischemia and reperfusion induced protein expression in NMCC within stained quadrants (Q1–Q3) in comparison with control cells (*n*=4) represented as a table.

Supplementary material Supplementary Fig. 2**.** (A)–(C) Representative FACS analysis data of NMCC following I/R induction along with live–dead assay. In the horizontal axis FITC labeled anti-Calp antibodies and for vertical axis PE labeled antibodies against Hsp90 were detected. Rest of the figures in the panel are derived from the quadrants of (A)–(C) and demonstrate the live–dead assay using 7-AAD. The studied conditions were; normal untreated NMCC (A); NMCC maintained in nutrient deficient buffer (ischemia induction) for 2 h (B); NMCC grown for 2 h in standard growth media containing 1 mM H_2_O_2_ subsequent to 2 h of ischemia induction (reperfusion induction) (C). (D) Histographical representation of comparative protein expression in ischemia and reperfusion induced NMCC with those of normal untreated NMCC within stained quadrants (Q1 – Hsp90; Q2 – Hsp90+Calp; Q3 – Calp) represented as fold level change (*n*=4). The fold level changes (increase or decrease) of protein expressing NMCC in each quadrant has been represented. Standard error was calculated and represented as error bars. (E) Fold level changes in ischemia and reperfusion induced protein expression in NMCC within stained quadrants (Q1–Q3) in comparison with control cells (*n*=4) represented as a table.

Supplementary material Supplementary Fig. 3. (A)–(C) Representative FACS analysis data of NMCC following I/R induction along with live–dead assay. In the horizontal axis FITC labeled anti-SarcAct antibodies and for vertical axis PE labeled antibodies against Hsp70 were detected. Rest of the figures in the panel are derived from the quadrants of (A)–(C) and demonstrate the live–dead assay using 7-AAD. The studied conditions were; normal untreated NMCC (A); NMCC maintained in nutrient deficient buffer (ischemia induction) for 2 h (B); NMCC grown for 2 h in standard growth media containing 1 mM H_2_O_2_ subsequent to 2 h of ischemia induction (reperfusion induction) (C). (D) Histographical representation of comparative protein expression in ischemia and reperfusion induced NMCC with those of normal untreated NMCC within stained quadrants (Q1 – Hsp70; Q2 – Hsp70+ SarcAct; Q3 – SarcAct) represented as fold level change (*n*=3). The fold level changes (increase or decrease) of protein expressing NMCC in each quadrant has been represented. Standard error was calculated and represented as error bars. (E) Fold level changes in ischemia and reperfusion induced protein expression in NMCC within stained quadrants (Q1–Q3) in comparison with control cells (*n*=3) represented as a table.

Supplementary material Supplementary Fig. 4. (A)–(C) Representative FACS analysis data of NMCC following I/R induction along with live–dead assay. In the horizontal axis FITC labeled anti-SarcAct antibodies and for vertical axis PE labeled antibodies against Hsp90 were detected. Rest of the figures in the panel are derived from the quadrants of the Supplementary Fig. 1(A)–(C) and demonstrate the live–dead assay using 7-AAD. The studied conditions were; normal untreated NMCC (A); NMCC maintained in nutrient deficient buffer (ischemia induction) for 2 h (B); NMCC grown for 2 h in standard growth media containing 1 mM H_2_O_2_ subsequent to 2 h of ischemia induction (reperfusion induction) (C). (D) Histographical representation of comparative protein expression in ischemia and reperfusion induced NMCC with those of normal untreated NMCC within stained quadrants (Q1 – Hsp90; Q2 – Hsp90+ SarcAct; Q3 – SarcAct) represented as fold level change (*n*=3). The fold level changes (increase or decrease) of protein expressing NMCC in each quadrant has been represented. Standard error was calculated and represented as error bars. (E) Fold level changes in ischemia and reperfusion induced protein expression in NMCC within stained quadrants (Q1–Q3) in comparison with control cells (*n*=3) represented as a table.

## Footnotes

Appendix A Supplementary data associated with this article can be found in the online version at doi:10.1016/j.bbrep.2015.09.016.
