# Supplementary material for: Expression of calcineurin, calpastatin and heat shock proteins during ischemia and reperfusion
Source: Biochem Biophys Rep. 2015 Sep 25;4:207–14. doi: 10.1016/j.bbrep.2015.09.016 (PMC6189699; doi:10.1016/j.bbrep.2015.09.016)
Supplement: Supplementary file 1 — Supplementary material Supplementary Fig. 1. (A)–(C) Representative FACS analysis data of NMCC following I/R induction along with live-dead assay. In the horizontal axis FITC labeled anti-Calp antibodies and for vertical axis PE labeled antibodies against Hsp70 were detected. Rest of the figures in the panel are derived from the quadrants of (A)–(C) and demonstrate the live-dead assay using 7-AAD. The studied conditions were; normal untreated NMCC (A); NMCC maintained in nutrient deficient buffer (ischemia induction) for 2 h (B); NMCC grown for 2 h in standard growth media containing 1 mM H2O2 subsequent to 2 h of ischemia induction (reperfusion induction) (C). (D) Histographical representation of comparative protein expression in ischemia and reperfusion induced NMCC with those of normal untreated NMCC within stained quadrants (Q1 – Hsp70; Q2 – Hsp70+Calp; Q3 – Calp) represented as fold level change (n=4). The fold level changes (increase or decrease) of protein expressing NMCC in each quadrant has been represented and significant values (p-value<0.05) denoted as *. Standard error was calculated and represented as error bars. (E) Fold level changes in ischemia and reperfusion induced protein expression in NMCC within stained quadrants (Q1–Q3) in comparison with control cells (n=4) represented as a table. [file mmc1.doc]

**Supplementary Materials - Table 1** : Antibodies and their dilutions used for FACS analysis

| No. | **Antibody** | **Dilution** |
| --- | --- | --- |
| **Primary antibodies** | | |
| **1.** | Mouse anti mouse heat shock protein 70 monoclonal antibodies (Sigma) | 1:500 |
| **2.** | Mouse anti mouse heat shock protein 90 monoclonal antibodies (Sigma) | 1: 50 |
| **3.** | Rabbit anti mouse α-sarcomeric actin polyclonal antibodies (Thermo Sci.) | 1:100 |
| **4.** | Rabbit anti human calpastatin polyclonal antibodies (Thermo Sci.) | 1:100 |
| **5.** | Rabbit anti bovine calcineurin polyclonal antibodies (in house - crude) | 1:50 |
|  |  |  |
| **Secondary antibodies** | | |
| **1.** | Goat antimouse IgG/M/A polyclonal antibodies – Alexa Flour 488 conjugated (Thermo Sci.) | 1: 2000 |
| **2.** | Goat anti rabbit IgG polyclonal antibodies – PE conjugated (Thermo Sci.) | 1: 1000 |
